# Does abnormal weight affect sperm quality? A case-control study based on bioelectrical impedance analysis

**DOI:** 10.3389/fnut.2025.1642836

**Published:** 2025-08-14

**Authors:** Huang Liu, Xiaoxia Wang, Li Li, Zhiyong Zhu, Hai Lin, Yu Zhou, Houbin Zheng

**Affiliations:** ^1^Department of Andrology, National Health Commission (NHC) Key Laboratory of Male Reproduction and Genetics, Guangdong Provincial Reproductive Science Institute (Guangdong Provincial Fertility Hospital), Human Sperm Bank of Guangdong Province, Guangzhou, China; ^2^Department of Immunology, National Health Commission (NHC) Key Laboratory of Male Reproduction and Genetics, Guangdong Provincial Reproductive Science Institute (Guangdong Provincial Fertility Hospital), Human Sperm Bank of Guangdong Province, Guangzhou, China; ^3^Department of Traditional Chinese Medicine, National Health Commission (NHC) Key Laboratory of Male Reproduction and Genetics, Guangdong Provincial Reproductive Science Institute (Guangdong Provincial Fertility Hospital), Human Sperm Bank of Guangdong Province, Guangzhou, China

**Keywords:** body composition, spermatogenesis, sperm parameters, bioelectrical impedance analysis, BMI

## Abstract

**Introduction:**

Weight gain can lead to metabolic, circulatory, and systemic changes. Obesity has been confirmed to induce various physical and mental illnesses. The relationship between abnormal weight and male fertility has become a research focus, although the findings regarding their correlation remain controversial.

**Objective:**

A case-control study based on bioelectrical impedance analysis was conducted to evaluate the correlation between abnormal body weight and sperm quality and to confirm the degree of impact of abnormal body weight on sperm quality.

**Methods:**

A retrospective analysis was conducted of 137 men who underwent fertility assessment at the Guangdong Provincial Reproductive Science Institute (Guangdong Provincial Fertility Hospital) between April 2024 and April 2025. Sperm parameters, body composition parameters, age, height, and other relevant information were extracted and analyzed. One hundred and thirty-seven males were divided into three groups according to their sperm quality: normal sperm group (Group A, *n* = 29), oligoasthenozoospermia group (Group B, *n* = 58), and azoospermia group (Group C, *n* = 50). According to the presence of sperm, they were divided into two groups: sperm group (AA group, *n* = 87) and azoospermia group (BB group, *n* = 50). The differences between these groups were compared, and the inherent connections and patterns between the indicators were explored through Pearson correlation analysis and partial correlation analysis; to determine the correlation between weight and sperm quality; and to evaluate the influence of weight on sperm quality.

**Results:**

The body composition parameters of the different sperm count groups (A, B, and C) were similar and showed no differences. However, there were certain differences in age, height, weight, protein (P), minerals (M), InBody score (IBS), percent body fat (PBF), total body water (TBW), intracellular water (ICW), body fat mass (BFM), body cell mass (BCM), soft lean mass (SLM), and skeletal muscle mass (SMM) between groups AA and BB. They did not follow a linear distribution, and the KMO and Bartlett sphericity tests suggested that they followed a spherical distribution (KMO = 0.775, sig = 0.000), which was related to the presence of sperm. Factor analysis revealed that weight, PBF, height, age, and IBS were the five key influencing factors. After combining height and weight factors, we found the age, IBS, and PBF were more decisive and sensitive than body mass index (BMI).

**Conclusion:**

Body composition has a certain impact on sperm quality, especially age, IBS, and PBF, which may be more accurate than BMI. Bioelectrical impedance analysis could effectively assist in the judgment and has the potential to predict sperm parameters.

## 1 Introduction

Obesity has become a global health concern, with numerous studies demonstrating its association with metabolic diseases, cardiovascular disorders, and musculoskeletal conditions (1–3). Emerging evidence suggests that obesity may also significantly affect human fertility, particularly in males (4–6). Although this viewpoint has not been confirmed by enough randomized controlled trials, clinical practice has found that the sperm parameters of some obese men were indeed significantly different from those of normal men (7, 8), while others seemed to have little relationship.

Studies have shown that obesity may reduce the biological activity of testosterone, leading to a decrease in free testosterone and inhibin B levels, an increase in 17 β-estradiol, and a decrease in sperm production ability (9). It may also regulate mast cells activity, increase the concentration of tumor necrosis factor, and inhibit growth (10). It may also lead to the aggregation of monocytes and macrophages, damage to the nucleus and mitochondrial DNA, and inhibition of sperm growth (11). Epidemiological investigations have found that children born to obese fathers have a significantly higher probability of developing obesity than normal individuals, and obesity may have an epigenetic impact on offspring (4, 12).

The main indicators used to evaluate male fertility worldwide are sperm parameters, including sperm concentration (C), total sperm count (A), percentage of forward-moving sperm (PR), percentage of normally shaped sperm (N), and sperm DNA fragmentation index (DFI). Studies have shown that in recent years, male sperm parameters have been declining annually (13, 14). This phenomenon has sounded the alarm for men around the world, and male reproductive health has become an increasingly hot research topic. Although there are still many different views on the relationship between obesity and male fertility internationally, as reproductive urologists, we believe it is our responsibility to guide patients in regulating their bodies correctly, avoiding harmful factors, and making every effort to protect fertility.

Body mass index (BMI) is an important index for evaluating obesity and is associated with the occurrence of many diseases. There have also been reports suggesting that BMI is related to sperm parameters (6). However, we have found in clinical practice that many men with high BMI still have ideal sperm parameters, while the quality of sperm with low BMI is not optimistic. Is BMI suitable for evaluating sperm quality? Is there an ideal indicator? Therefore, to correctly evaluate the correlation between abnormal weight and sperm quality; and to confirm the degree of influence of obesity on sperm quality, we conducted a case-control study based on bioelectrical impedance analysis.

## 2 Data and methods

### 2.1 Clinical data

Under the principle of informed consent, we enrolled 137 male patients undergoing fertility assessment at the Guangdong Provincial Reproductive Science Institute (Guangdong Provincial Fertility Hospital) between April 2024 and April 2025. They were aged between 23 and 54 years. Their height was between 160 and 186 cm. Each group was monitored for sperm parameters, body composition, reproductive hormones, and other indicators. Each patient's demographic characteristics, such as age and fertility status, were also recorded in detail. The indicators included sperm concentration (C), total sperm count (A), forward motile sperm percentage (PR), normal form sperm percentage (N), DFI, protein (P), minerals (M), body mass index (BMI), InBody score (IBS), fat composition [containing percent body fat (PBF), body fat mass (BFM), fat-free mass (FFM), and fat mass index (FMI)], water composition [containing total body water (TBW), intracellular water (ICW), and extracellular water (ECW)], and mass proportion [containing body cell mass (BCM), soft lean mass (SLM), and skeletal muscle mass (SMM)]. According to the quality of sperm, they were divided into the following three groups: normal sperm group (C ≧ 15 × 10^6^/ml, A ≧ 39 × 10^6^/ejaculation, PR ≧ 32%, *N* ≧ 4%; Group A, *n* = 29), and oligoasthenozoospermia group (0 <C <15 × 10^6^/ml, 0 <A <39 × 10^6^/ejaculation, 0 <PR <32%, 0 <N <4%; Group B, *n* = 58), and azoospermia group (C = 0 × 10^6^/ml, A = 0 × 10^6^/ejaculation, PR = 0, N = 0, no sperm were found in the semen after centrifugation; Group C, *n* = 50). According to the presence of sperm, they were also divided into two groups: one was the sperm group (AA group, *n* = 87) and the other group was the azoospermia group (BB group, *n* = 50).

#### 2.1.1 Inclusion criteria

(1) Willingness to participate; (2) normal mobility; (3) ability to provide semen samples via masturbation; (4) no fertility treatments in the preceding 3 months; and (5) 2–7 days of abstinence.

#### 2.1.2 Exclusion criteria

(1) People with implanted medical devices, such as pacemakers or their basic supporting equipment (such as patient monitoring systems), inside their bodies are not allowed to use this device. During the testing process, a safe low current may flow through the human body, which may cause such equipment to malfunction or pose a threat to life safety. (2) People with limited mobility need to be supervised and assisted by others; therefore, they were excluded. (3) People with infectious diseases were excluded because they may pollute the InBody equipment.

### 2.2 Testing methods

#### 2.2.1 Semen testing

##### 2.2.1.1 Semen characteristics

According to the WHO standards (15, 16), male participants were instructed to use masturbation to collect fresh ejaculated semen within 2–7 days of abstinence. The volume of semen was measured by weighing, and the pH of the semen was measured using precision pH test strips. The collected semen was placed in a non-toxic wide-mouthed bottle and fully liquefied in a 37°C water bath. After stirring evenly, 10 μl of the sample was taken and sent for microscopic examination (Olympus, Germany). If no sperm was found in fresh semen by microscopy, the semen was transferred to a centrifuge tube and centrifuged at 3,000 g/min for 15 min. The centrifuged sediment was used for microscopic examination, and the total number of sperm ejaculated was calculated each time. The operation method refers to the relevant standards of the WHO Laboratory Manual for Human Semen Examination and Processing (referred to as the Manual; 5th edition) (15), and is processed and tested by a dedicated individual. Semen characteristics include analysis of color, semen volume, viscosity, pH value, and non-sperm cell count.

##### 2.2.1.2 Sperm parameters

According to the relevant standards of the WHO Laboratory Manual for Human Semen Examination and Treatment (referred to as the Manual) (15, 16), a dedicated person is responsible for detecting sperm concentration (C), total sperm count (A), percentage of forward-moving sperm (PR), and percentage of normally shaped sperm (N). The total number of sperm ejaculated each time (A) = sperm concentration (C) × semen volume (V).

##### 2.2.1.3 Sperm DFI

A BD flow cytometer (FACSCanto II) was used to detect the sperm DNA integrity index (DFI), and the detection reagent used was acridine orange staining solution (registration number: Yueshen Food and Drug Supervision Equipment Production Preparation No. 20150071, Shenzhen Huakang Biomedical Engineering Co., Ltd.). The experimental process was strictly followed according to the reagent instructions. After acid treatment of the semen sample, the damaged sperm nuclear DNA did not undergo oxidation of the fish sperm protein SH, resulting in a loose chromatin structure and the formation of single strands, which were detected using flow cytometry. The chromatin of damaged sperm nuclei becomes single stranded, and after binding with acridine orange, it is excited by a 488 nm laser to produce red or yellow fluorescence. The chromatin of normal sperm nuclei can maintain a complete double-stranded structure and bind with acridine orange to produce green fluorescence. An increases in the proportion of red-light sperm indicates an increase in the degree of damage to sperm nuclear integrity. At least 10,000 sperm for analysis, and the sperm DNA fragmentation index (DFI) was calculated based on the fluorescence signal intensity. Reference value for DFI: normal: ≤ 15%, abnormal: >15%.

##### 2.2.1.4 Reproductive hormones

Peripheral venous blood was collected from the men in their abdominal state at 8:00 a.m. After settling, the serum and red blood cells were separated. The Elecsys2010 fully automatic electrochemiluminescence immunoassay analyzer was used to detect the concentrations of follicle stimulating hormone (FSH), luteinizing hormone (LH), prolactin (PRL), and testosterone (T) in the serum, and inhibin B (INHb) was detected by ELISA. The entire process was strictly performed according to the SOP document. Quality control within or between batches was performed using the quality control products carried by the testing instrument itself. Reference value for FSH: 0.95–11.95 mIU/ml; the reference values of LH: 0.57–12.07 mIU/ml; reference value of PRL: 72.66–407.4 mIU/L; reference value for T: 4.94–32.01 nmol/L; reference value for INHb: 21–368 pg/ml.

#### 2.2.2 Body composition testing

**Figure 1:**
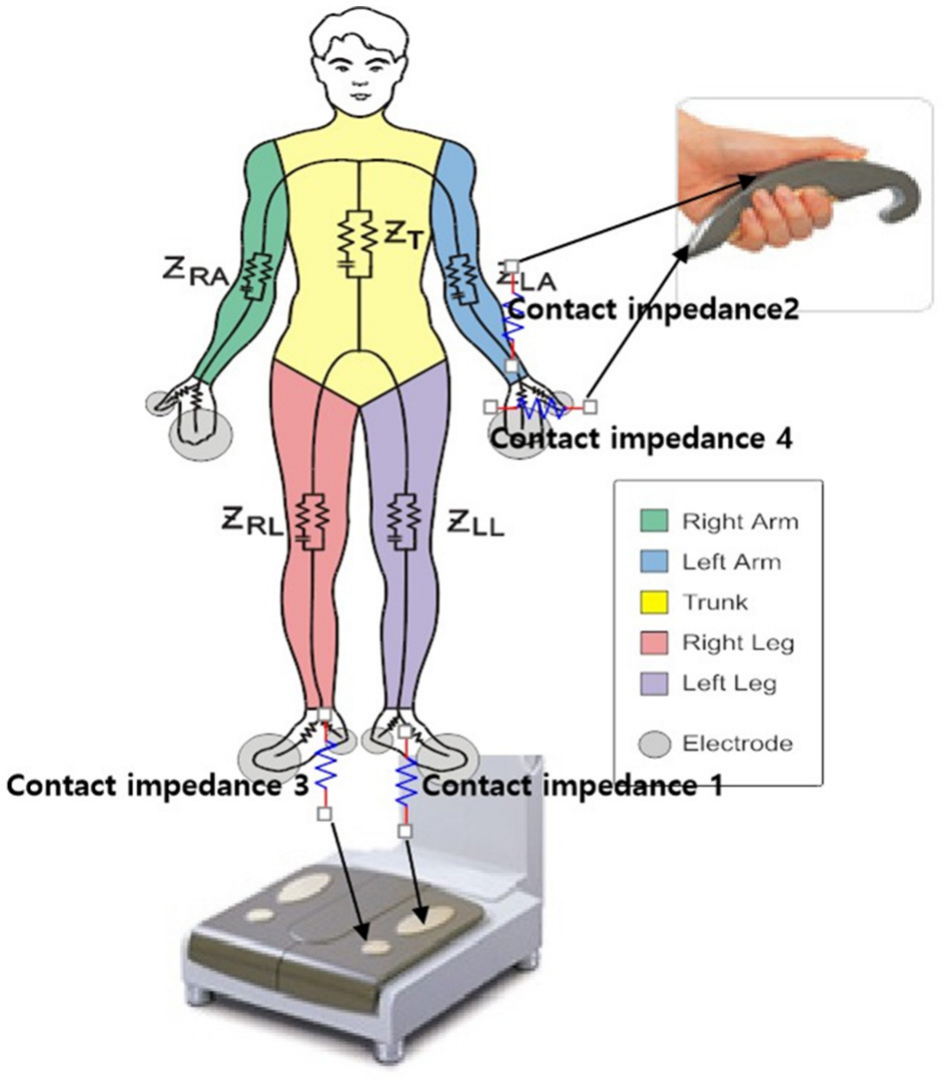
Schematic diagram of bioelectrical impedance detection.

Body composition was detected using an InBody770 bioelectrical impedance analyzer (Biospace Building, 54, Nonhyeon-ro 2-gil, Gangnam gu, Seoul 135-960 KOREA), with room temperature maintained at 26°C and humidity maintained at 70%. A dedicated person was responsible for the entire process, and the report was verified by two doctors (Figure 1). The men were in an empty stomach state and stood for 5 min before the test. If the man had already eaten before the test, the test was postponed to 2 h after meals. It was recommended that the man used the toilet and avoided physical exercise before testing, as feces or exercise would cause temporary changes in body composition, affecting the accuracy of the results. It was recommended to test men at breakfast and clean the excessively dry or thick stratum corneum on the palms and soles of the feet to avoid increasing the difficulty of the test. The observation indicators include: protein (P), minerals (M), body mass index (BMI), InBody score (IBS), fat composition [containing percent body fat (PBF), body fat mass (BFM), fat-free mass (FFM), and fat mass index (FMI)], Water composition [containing total body water (TBW), intracellular water (ICW), extracellular water (ECW)], and mass proportion [containing body cell mass (BCM), soft lean mass (SLM), and skeletal muscle mass (SMM)], with a total of 14 indicators.


$$\begin{array}{l} BMI\;\left(Body\;Mass\;index\right)=\frac{height\;\left(cm\right)}{{weight\;\left(kg\right)}^{2}} \end{array}$$

### 2.3 Statistical methods

SPSS software (version 19.0; SPSS Inc., Chicago) was used for statistical analysis. The K-S test and homogeneity of variation test are used for normality testing and intergroup analysis. Pearson correlation analysis and partial correlation analysis were used to determine the relationship between various indicators. Univariate analysis of variance, analysis of variance (ANOVA), multivariate analysis of covariance (MANOVA), factor analysis and KMO, and Bartlett sphericity tests were used to determine subject effects and inter-group effects. Statistical significance was set at *p* < 0.05 and a critical value of ±1.96.

### 2.4 Ethical statement

This project was approved by the ethics committee of the Guangdong Provincial Reproductive Science Institute (Guangdong Provincial Fertility Hospital; approval no. [2025] (9).

## 3 Results

The ages of the 137 men ranged from 23 to 54 years, with an average of 32.92 ± 5.68 years. The height was between 160 and 186 cm, with an average of 172.27 ± 5.60 cm. The weight ranged from 50.5 to 118.2 kg, with an average of 76.25 ± 12.99 kg.

### 3.1 Analysis of general demographic characteristics

ANOVA showed no significant differences in age and height among males in groups A, B, and C (*p* > 0.05). There was also no difference in age and height between the AA and BB groups (*p* > 0.05; Table 1).

**Table 1:** General demographic characteristics.

**Groups**	**Sperm quality**					**Sperm presence**			
	**A**	**B**	**C**	**F**	***p***	**AA**	**BB**	***t***	***p***
Size	29	58	50	–	–	87	50	–	–
Age (years)	32.55 ± 4.90	34.22 ± 6.45	31.62 ± 4.93	2.958	0.055	33.67 ± 6.001	31.62 ± 4.93	2.046	0.05
Height (cm)	171.52 ± 5.58	172.38 ± 5.81	172.58 ± 5.62	0.343	0.710	172.09 ± 5.72	172.58 ± 5.50	−0.493	0.627

### 3.2 Normality test

K-S inspection prompt: all 13 indicators are equal and follow a normal distribution, except for IBS (Table 2).

**Table 2:** Normality distribution of indicators.

**Indicator**	**Levin-value**	**df 1**	**df 2**	***p***
P	0.258	2	134	0.773
M	0.288	2	134	0.750
BMI	0.280	2	134	0.756
IBS	3.853	2	134	0.024^*^
PBF	2.016	2	134	0.137
TBW	0.265	2	134	0.768
ICW	0.324	2	134	0.724
ECW	0.130	2	134	0.878
BFM	1.523	2	134	0.222
BCM	0.333	2	134	0.718
SLM	0.275	2	134	0.760
FFM	0.287	2	134	0.751
SMM	0.313	2	134	0.732
FMI	1.346	2	134	0.264

^*^p < 0.05.

P, protein; M, minerals; BMI, body mass index; IBS, InBody score; PBF, percent body fat; TBW, total body water; ICW, intracellular water; ECW, extracellular water; BFM, body fat mass; BCM, body cell mass; SLM, soft lean mass; FFM, fat free mass; SMM, skeletal muscle mass; FMI, fat mass index.

### 3.3 Relevant analysis

Pearson correlation analysis suggested that the 14 indicators were not related to the total sperm count (*p* > 0.05). After controlling for age and height, partial correlation analysis suggested that the 14 indicators were also not related to the total sperm count (*p* > 0.05; Table 3).

**Table 3:** The relationship between total sperm number and indicators.

**Control**		**A (n=137)**	**P**	**M**	**BMI**	**IBS**	**PBF**	**TBW**	**ICW**	**ECW**	**BFM**	**BCM**	**SLM**	**FFM**	**SMM**	**FMI**
	None	*r*	0.070	0.065	0.009	0.040	0.007	0.064	0.068	0.056	0.009	0.068	0.065	0.065	0.068	−0.008
		*p*	0.419	0.454	0.920	0.640	0.939	0.457	0.428	0.515	0.918	0.430	0.451	0.451	0.429	0.930
	Age and height	*r*	0.075	0.071	0.007	0.047	0.001	0.069	0.074	0.061	0.006	0.074	0.070	0.070	0.074	−0.012
		*p*	0.385	0.415	0.935	0.588	0.992	0.425	0.393	0.468	0.947	0.395	0.418	0.417	0.395	0.892

A, Total sperm count; P, protein; M, minerals; BMI, body mass index; IBS, InBody score; PBF, percent body fat, TBW, total body water; ICW, intracellular water; ECW, extracellular water; BFM, body fat mass; BCM, body cell mass; SLM, soft lean mass; FFM, fat free mass; SMM, skeletal muscle mass; FMI, fat mass index.

### 3.4 Multivariate covariance analysis

**Figure 2:**
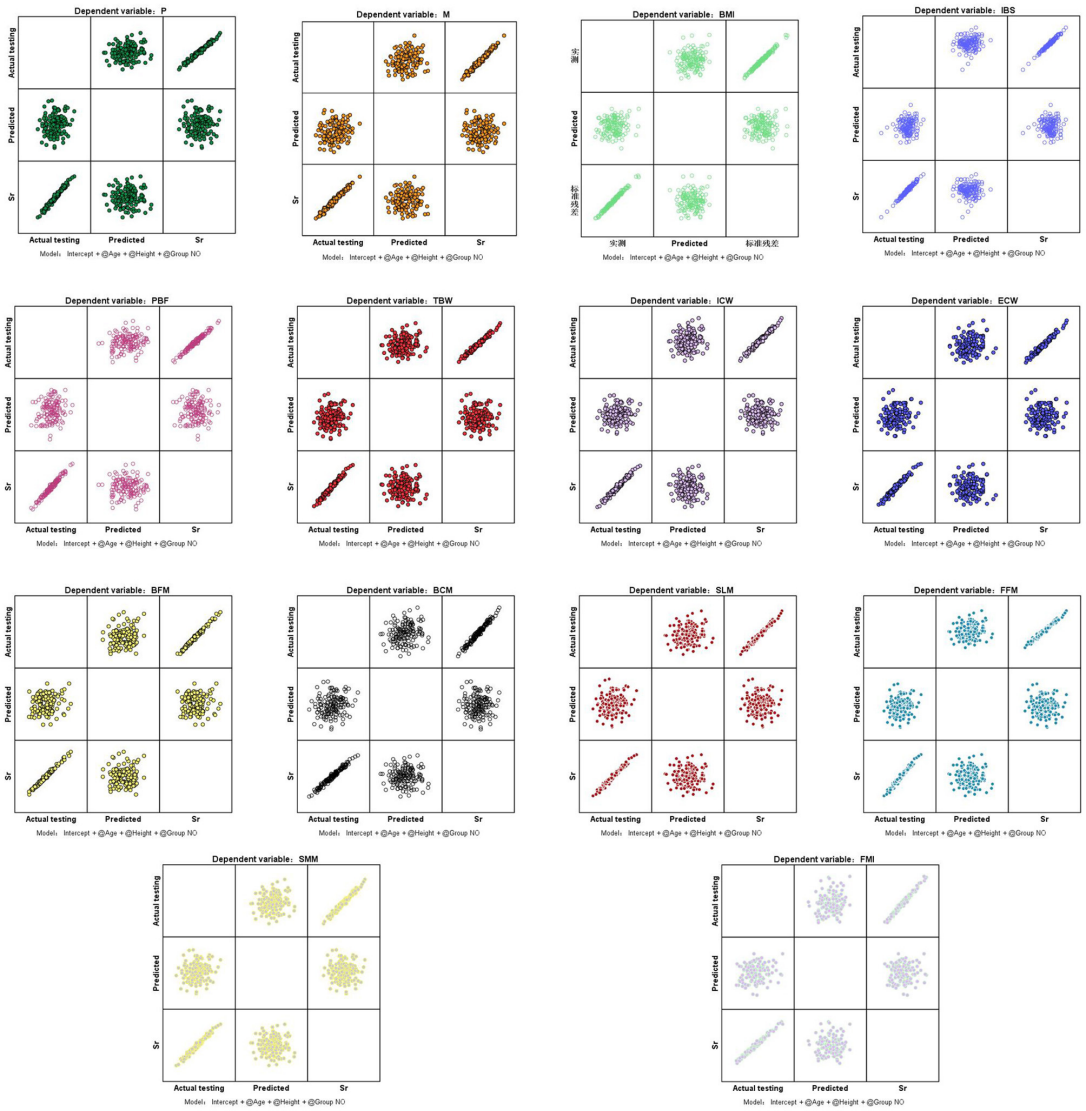
Standardized residual plots of indicators. P, protein; M, minerals; BMI, body mass index; IBS, InBody score; PBF, percent body fat; TBW, total body water; ICW, intracellular water; ECW, extracellular water; BFM, body fat mass; BCM, body cell mass; SLM, soft lean mass; FFM, fat free mass; SMM, skeletal muscle mass; FMI, fat mass index. Sr, standardized residual.

Multivariate analysis of variance (MANOVA) showed that after controlling for age and height, there was no difference in the effect test among the 14 indicators in groups A, B, and C (*p* > 0.05; Table 4, Figure 2).

**Table 4:** Partial correlation analysis between total sperm number and indicators.

**Model**	**Dependent variable**	**Type III SS**	**df**	**ms**	***F***	**Sig**	**SD of Eta**
Error correction model	P	7.396	4	1.849	0.954	0.435	0.028
	M	1.054	4	0.263	1.040	0.389	0.031
	BMI	28.531	4	7.133	0.435	0.783	0.013
	IBS	117.966	4	29.491	0.585	0.674	0.017
	PBF	186.960	4	46.740	0.873	0.482	0.026
	TBW	102.589	4	25.647	0.973	0.425	0.029
	ICW	38.859	4	9.715	0.924	0.452	0.027
	ECW	15.643	4	3.911	1.071	0.373	0.031
	BFM	137.102	4	34.275	0.488	0.744	0.015
	BCM	78.855	4	19.714	0.914	0.458	0.027
	SLM	168.269	4	42.067	0.964	0.429	0.028
	FFM	190.998	4	47.749	0.972	0.425	0.029
	SMM	65.707	4	16.427	0.921	0.454	0.027
	FMI	22.292	4	5.573	0.712	0.585	0.021

P, protein; M, minerals; BMI, body mass index; IBS, InBody score; PBF, percent body fat, TBW, total body water; ICW, intracellular water; ECW, extracellular water; BFM, body fat mass; BCM, body cell mass; SLM, soft lean mass; FFM, fat free mass; SMM, skeletal muscle mass; FMI, fat mass index.

### 3.5 Standardization analysis and factor analysis

**Figure 3:**
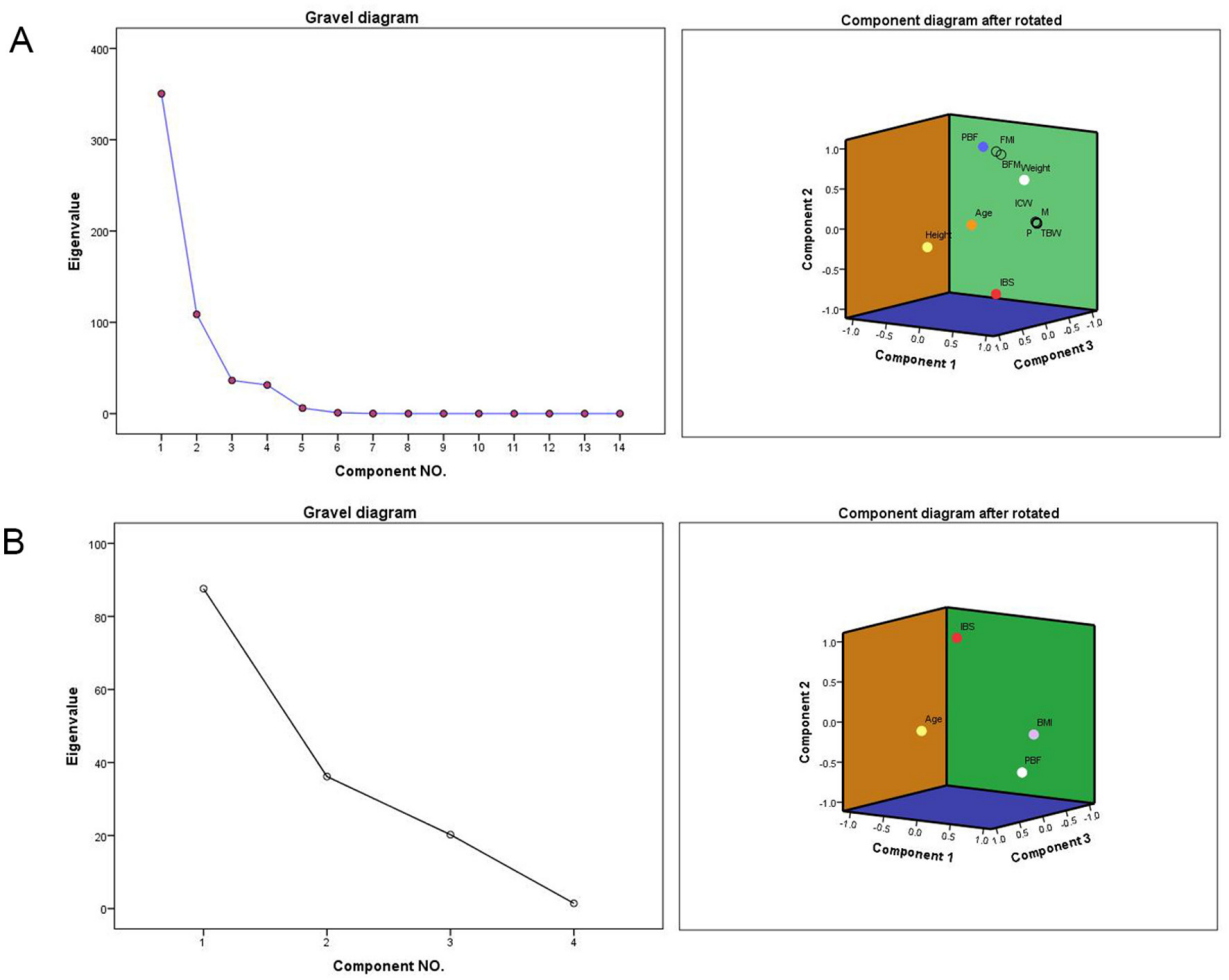
Rotating Component Matrix and Scree Plot. P, protein; M, minerals; IBS, InBody score; PBF, percent body fat; TBW, total body water; ICW, intracellular water; BFM, body fat mass; BCM, body cell mass; SLM, soft lean mass; SMM, skeletal muscle mass; FMI, fat mass index. **(A)** Gravel diagram after extracting 5 factors and the Component Matrix diagram after rotation. **(B)** Gravel diagram after extracting 3 factors and the Component Matrix diagram after rotation.

After standardizing all data, the KMO and Bartlett sphericity tests were applied to determine whether the variances of the AA groups of data were equal. The results showed a significant difference between patients in terms of age, height, weight, P, M, IBS, PBF, TBW, ICW, BFM, BCM, SLM, and SMM. Factor analysis identified five key components influencing sperm presence: weight, percent body fat (PBF), height, age, and InBody score (IBS; KMO = 0.775, *p* < 0.001). When combining height and weight factors, age, IBS, and PBF emerged as more sensitive discriminators than BMI alone (Tables 5A–C, Figure 3).

**Table 5A:** The distribution trend of sphericity for indicators.

**Indicator**	**Bartlett sphericity test**	**KMO test**
Approximate *χ^2^*	5,461.608	–
df	91	–
Sig	0.000^**^	0.775

^**^p<0.001.

**Table 5B:** The structural distribution of related factors after rotation.

**Indicator**	**Original composition**					**Re scale the components after rotation**				
	**1**	**2**	**3**	**4**	**5**	**1**	**2**	**3**	**4**	**5**
Age	−0.075	0.001	−0.126	6.003	−0.020	−0.012	0.000	−0.021	1.000	−0.003
Height	0.180	−0.727	5.665	−0.123	−0.016	0.031	−0.127	0.991	−0.021	−0.003
Weight	10.209	8.465	−0.447	−0.065	0.044	0.769	0.638	−0.034	−0.005	0.003
P	1.372	0.176	0.016	−0.004	0.061	0.989	0.127	0.011	−0.003	0.044
M	0.478	0.069	−0.009	−0.015	0.019	0.949	0.137	−0.018	−0.029	0.038
IBS	2.386	−5.535	−0.122	−0.184	2.921	0.356	−0.826	−0.018	−0.027	0.436
PBF	0.870	6.796	−0.494	−0.028	0.539	0.126	0.981	−0.071	−0.004	0.078
TBW	5.055	0.698	0.066	0.007	0.098	0.990	0.137	0.013	0.001	0.019
ICW	3.195	0.397	0.030	−0.019	0.133	0.990	0.123	0.009	−0.006	0.041
BFM	3.302	7.521	−0.522	−0.050	−0.126	0.400	0.912	−0.063	−0.006	−0.015
BCM	4.574	0.573	0.037	−0.025	0.190	0.990	0.124	0.008	−0.005	0.041
SLM	6.507	0.884	0.080	−0.003	0.157	0.990	0.135	0.012	0.000	0.024
SMM	4.159	0.520	0.036	−0.022	0.176	0.990	0.124	0.009	−0.005	0.042
FMI	0.840	2.574	−0.258	−0.017	0.202	0.306	0.937	−0.094	−0.006	0.074

P, protein; M, minerals; IBS, InBody score; PBF, percent body fat; TBW, total body water; ICW, intracellular water; BFM, body fat mass; BCM, body cell mass; SLM, soft lean mass; SMM, skeletal muscle mass; FMI, fat mass index.

**Table 5C:** The structural distribution of related factors before rotation.

**Indicator**	**Component score coefficient**				
	**1**	**2**	**3**	**4**	**5**
Age	0.001	0.005	0.022	1.001	0.070
Height	−0.022	0.076	1.020	0.022	0.198
Weight	0.428	0.276	0.007	−0.001	−0.101
P	0.012	−0.006	−0.001	0.000	−0.014
M	0.001	−0.001	0.000	0.000	−0.002
IBS	−0.008	−0.050	−0.005	0.005	2.117
PBF	−0.266	0.454	0.092	0.010	1.364
TBW	0.169	−0.097	−0.014	0.004	−0.287
ICW	0.064	−0.036	−0.005	0.000	−0.081
BFM	−0.103	0.380	0.038	−0.005	0.525
BCM	0.132	−0.073	−0.012	0.001	−0.166
SLM	0.277	−0.157	−0.023	0.006	−0.444
SMM	0.109	−0.060	−0.009	0.001	−0.135
FMI	−0.029	0.060	0.005	0.001	0.178

P, protein; M, minerals; IBS, InBody score; PBF, percent body fat; TBW, total body water; ICW, intracellular water; BFM, body fat mass; BCM, body cell mass; SLM, soft lean mass; SMM, skeletal muscle mass; FMI, fat mass index.

## 4 Discussion

The change of body composition structure is the best embodiment of body metabolism (17). Research showed that: different compositions of protein, fat and water would lead to significant differences in the growth ability and resistance of the body, and there were significant differences between the body composition of patients with tumors, diabetes and healthy people (18–20). The existence of the blood testis barrier provided a natural guarantee for the occurrence and maturation of sperm, but this barrier could also rupture due to abnormal metabolism in the body. Research had shown that after the disruption of the blood testis barrier, the occurrence of sperm would significantly decrease, and even leaded to the occurrence of azoospermia (21, 22). However, was there a correlation between these phenomena and structural changes in body composition? There was currently no relevant research available. Based on this, ours study used bioelectrical impedance analysis to determine the correlation between body structural components and sperm parameters, which could provide evidence-based medicine evidence to support the correlation between changes in body composition and sperm quality.

Age and aging are inevitable factors in the decline of human function, and the production and maturation of sperm also have the same experience. Research had shown that with age, the parameters of male sperm would significantly decrease (23). But there was also data showing that although the sperm parameters of elderly men had decreased, the fertilization ability of sperm still remained relatively unchanged (24). Therefore, there had been much controversy in the industry regarding whether elderly men might experience a decline in fertility. We also found that in our research, before controlling of age, there were no difference between body composition indicators in the group A, group B, and group C of P, M, BMI, IBS, PBF, TBW, ICW, ECW, BFM, BCM, SLM, FFM, SMM, FMI, and the correlation between them and the occurrence of sperm was not high. This might be one of the reasons why individual differences were not significant among different age groups. After controlling of age and conducting precise analysis, we found that there were significant differences in eight out of 14 body composition indicators among the three groups. This result precisely confirmed that the greater of the structural differences in body composition among the same age group, the more significant the differences in sperm parameters might be, especially in indicators such as PBF and IBS, where the man with sperm was significantly lower than the man without sperm. Therefore, we highly suspected that abnormal increases in indicators such as weight, PBF, height and IBS at the same age might lead to the occurrence of azoospermia. In order to ensure better fertility, man should strictly control their weight and adjust their body composition structure.

PBF is a reflection of body fat content, and the impact of fat on sperm production function has been confirmed in our previous research and also confirmed by other studies (25, 26). BMI reflects the relationship between height and weight, but it seems to have no direct indication of the degree of fat accumulation in the body. Therefore, PBF has an advantage over BMI in evaluating total fat. IBS is an overall score of body composition, which comprehensively reflects indicators such as muscle mass, fat, and water content, reflecting the balance of human body structure. From its composition perspective, IBS is also more comprehensive and accurate than BMI. Therefore, based on the findings of this study, we believe that PBF and IBS should be able to replace BMI as more accurate body composition indicators for evaluating sperm parameters.

Preventing diseases has become the mainstream guiding ideology of modern medicine. With the change of health concepts, China had put forward significant measures to control weight, prevent diseases, and promote public health (27, 28). The improvement of male fertility may also benefit from weight control. Our research had found that abnormal body composition indicators were potential hazards to spermatogenesis. Through factor analysis, we extracted the main factors closely related to sperm development, among which age, IBS, and PBF were the top three main factors affecting sperm quality, and each of them had unique ways of affecting sperm quality. This might be the starting point for our treatment to improve male sperm quality. If we started from three major directions such as body composition and adjust their proportion structure, we believed it had reason to bring benefits to the improvement of sperm quality.

## 5 Conclusion

Our findings suggest that body composition significantly impacts sperm quality, with age, IBS, and PBF showing greater discriminatory power than BMI in predicting sperm presence. Bioelectrical impedance analysis may serve as a valuable tool for assessing male fertility potential.

## Data Availability

The datasets presented in this study can be found in online repositories. The names of the repository/repositories and accession number(s) can be found in the article/supplementary material.
